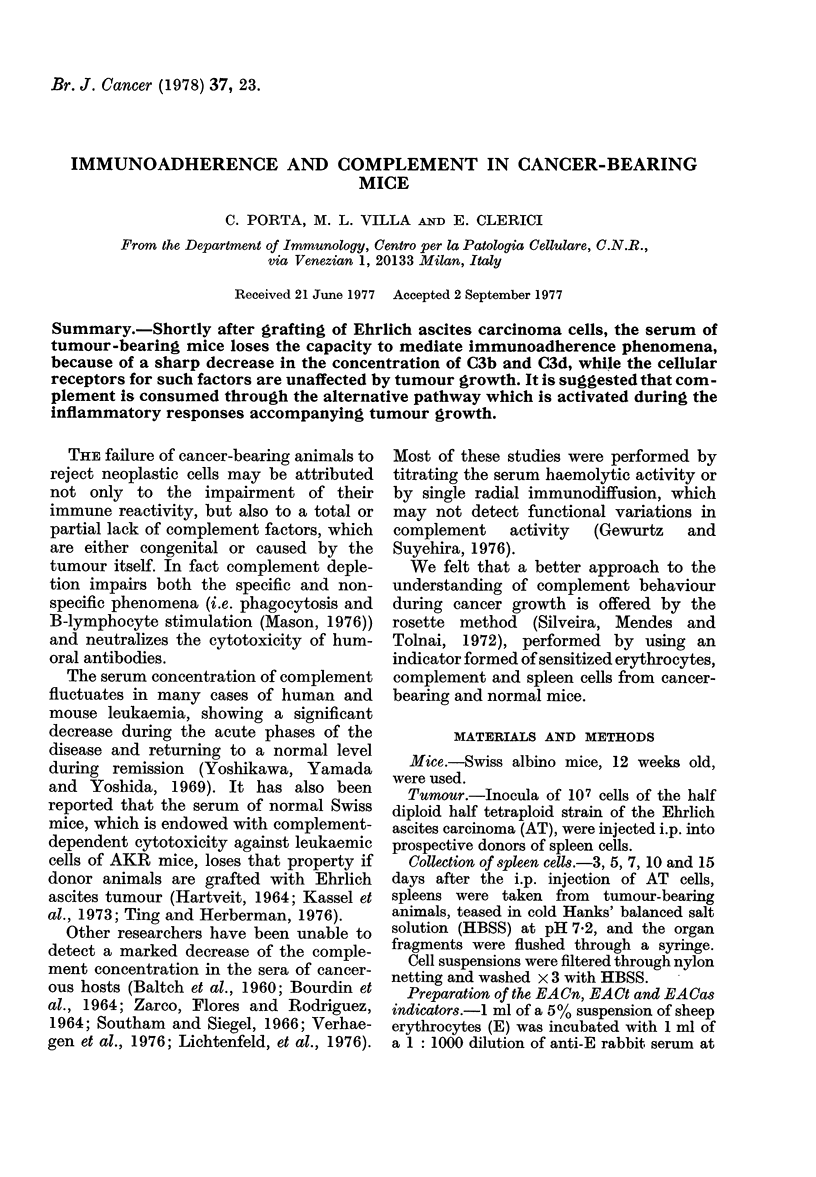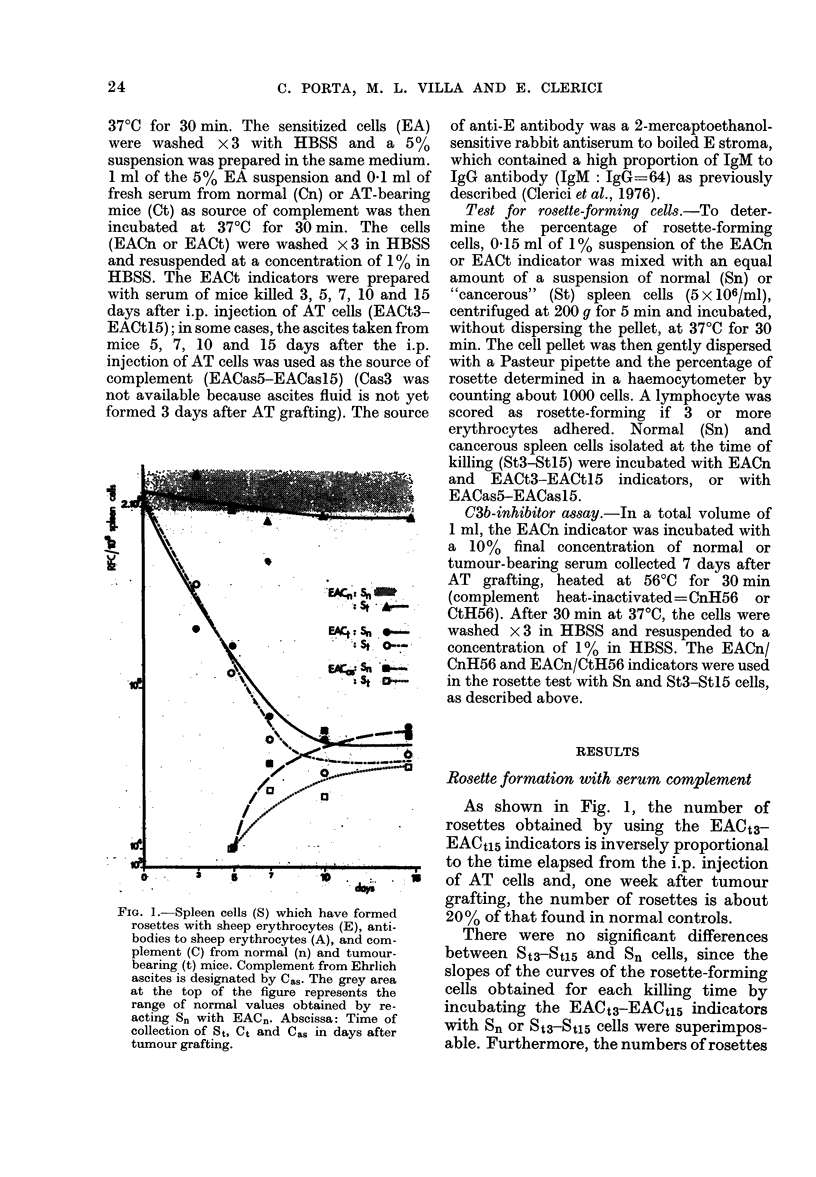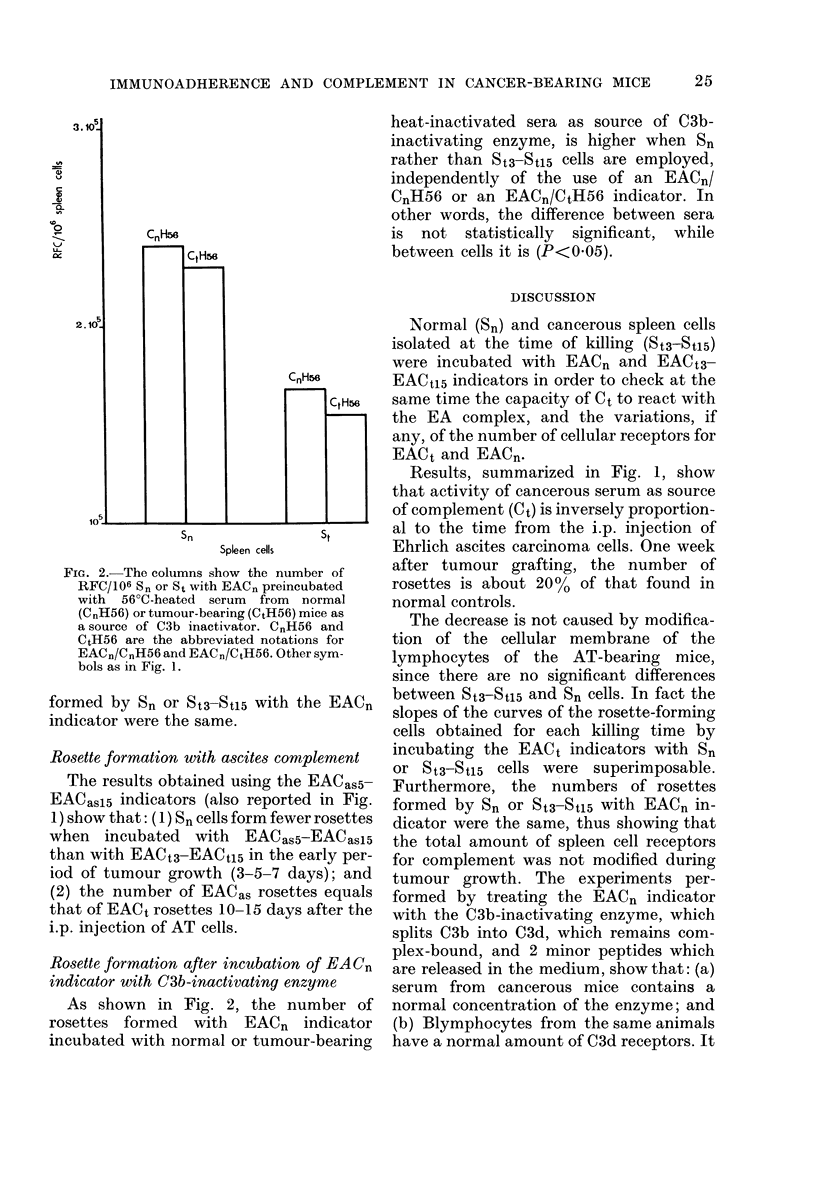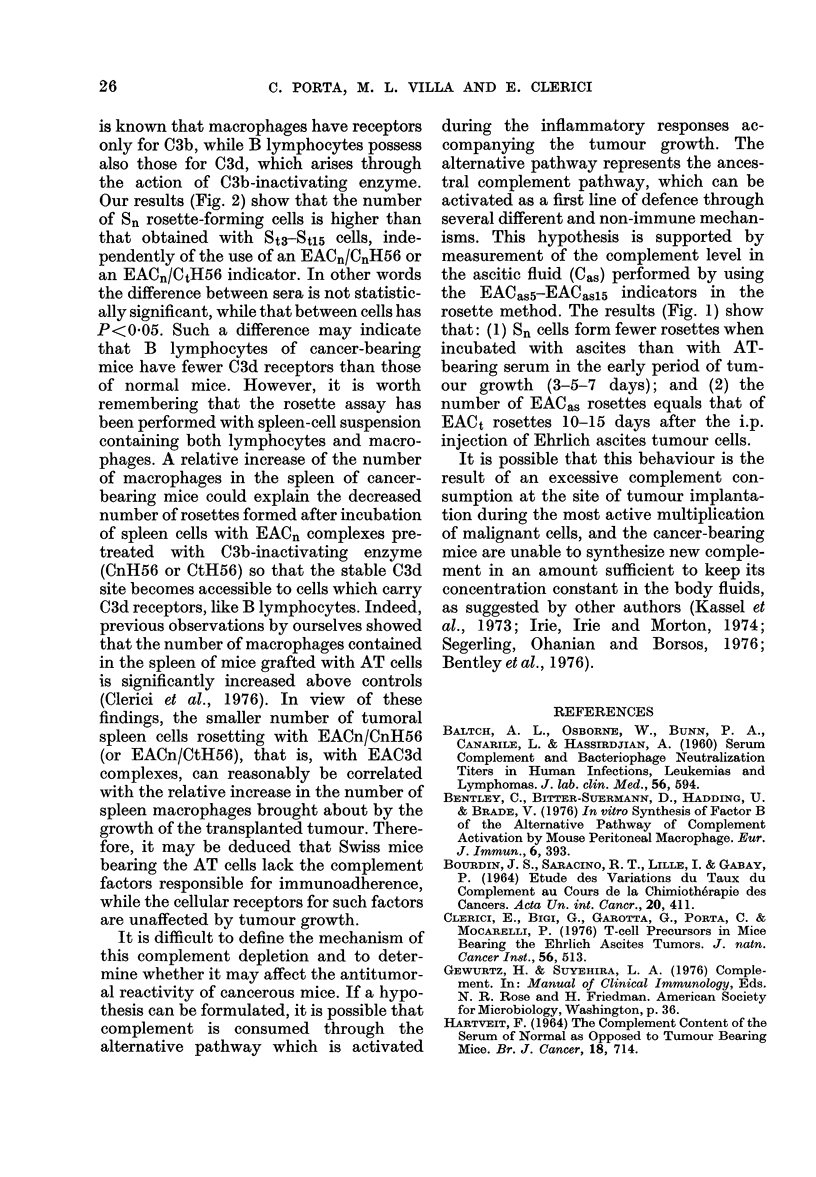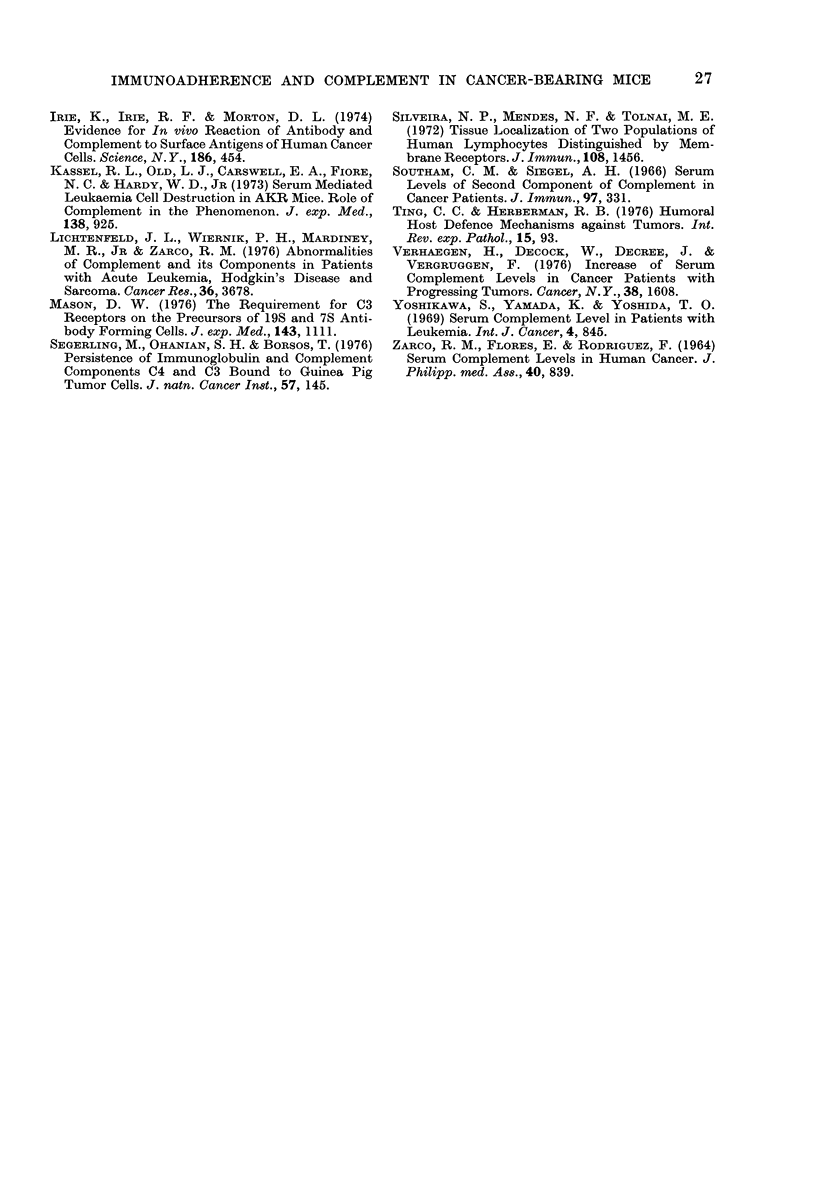# Immunoadherence and complement in cancer-bearing mice.

**DOI:** 10.1038/bjc.1978.3

**Published:** 1978-01

**Authors:** C. Porta, M. L. Villa, E. Clerici

## Abstract

Shortly after grafting of Ehrlich ascites carcinoma cells, the serum of tumour-bearing mice loses the capacity to mediate immunoadherence phenomena, because of a sharp decrease in the concentration of C3b and C3d, while the cellular receptors for such factors are unaffected by tumour growth. It is suggested that complement is consumed through the alternative pathway which is activated during the inflammatory responses accompanying tumour growth.


*Br. J. Cancer* (1978) **37**, 23.

### IMMUNOADHERENCE AND COMPLEMENT IN CANCER-BEARING MICE

C. PORTA, M. L. VILLA AND E. CLERICI

*From the Department of Immunology, Centro per la Patologia Cellulare, C.N.R., via Venezian* 1, 20133 *Milan, Italy*

Received 21 June 1977 Accepted 2 September 1977

**Summary.—Shortly after grafting of Ehrlich ascites carcinoma cells, the serum of tumour-bearing mice loses the capacity to mediate immunoadherence phenomena, because of a sharp decrease in the concentration of C3b and C3d, while the cellular receptors for such factors are unaffected by tumour growth. It is suggested that complement is consumed through the alternative pathway which is activated during the inflammatory responses accompanying tumour growth.**

THE failure of cancer-bearing animals to reject neoplastic cells may be attributed not only to the impairment of their immune reactivity, but also to a total or partial lack of complement factors, which are either congenital or caused by the tumour itself. In fact complement depletion impairs both the specific and non-specific phenomena (*i.e.* phagocytosis and B-lymphocyte stimulation (Mason, 1976)) and neutralizes the cytotoxicity of humoral antibodies.

The serum concentration of complement fluctuates in many cases of human and mouse leukaemia, showing a significant decrease during the acute phases of the disease and returning to a normal level during remission (Yoshikawa, Yamada and Yoshida, 1969). It has also been reported that the serum of normal Swiss mice, which is endowed with complement-dependent cytotoxicity against leukaemic cells of AKR mice, loses that property if donor animals are grafted with Ehrlich ascites tumour (Hartveit, 1964; Kassel *et al.*, 1973; Ting and Herberman, 1976).

Other researchers have been unable to detect a marked decrease of the complement concentration in the sera of cancerous hosts (Baltch *et al.*, 1960; Bourdin *et al.*, 1964; Zarco, Flores and Rodriguez, 1964; Southam and Siegel, 1966; Verhaegen *et al.*, 1976; Lichtenfeld, *et al.*, 1976). Most of these studies were performed by titrating the serum haemolytic activity or by single radial immunodiffusion, which may not detect functional variations in complement activity (Gewurtz and Suyehira, 1976).

We felt that a better approach to the understanding of complement behaviour during cancer growth is offered by the rosette method (Silveira, Mendes and Tolnai, 1972), performed by using an indicator formed of sensitized erythrocytes, complement and spleen cells from cancer-bearing and normal mice.

### MATERIALS AND METHODS

*Mice.*—Swiss albino mice, 12 weeks old, were used.

*Tumour.*—Inocula of $10^7$ cells of the half diploid half tetraploid strain of the Ehrlich ascites carcinoma (AT), were injected i.p. into prospective donors of spleen cells.

*Collection of spleen cells.*—3, 5, 7, 10 and 15 days after the i.p. injection of AT cells, spleens were taken from tumour-bearing animals, teased in cold Hanks' balanced salt solution (HBSS) at pH 7·2, and the organ fragments were flushed through a syringe. Cell suspensions were filtered through nylon netting and washed ×3 with HBSS.

*Preparation of the EACn, EACt and EACas indicators.*—1 ml of a 5% suspension of sheep erythrocytes (E) was incubated with 1 ml of a 1 : 1000 dilution of anti-E rabbit serum at

37°C for 30 min. The sensitized cells (EA) were washed ×3 with HBSS and a 5% suspension was prepared in the same medium. 1 ml of the 5% EA suspension and 0·1 ml of fresh serum from normal (Cn) or AT-bearing mice (Ct) as source of complement was then incubated at 37°C for 30 min. The cells (EACn or EACt) were washed ×3 in HBSS and resuspended at a concentration of 1% in HBSS. The EACt indicators were prepared with serum of mice killed 3, 5, 7, 10 and 15 days after i.p. injection of AT cells (EACt3–EACt15); in some cases, the ascites taken from mice 5, 7, 10 and 15 days after the i.p. injection of AT cells was used as the source of complement (EACas5–EACas15) (Cas3 was not available because ascites fluid is not yet formed 3 days after AT grafting). The source

FIG. 1.—Spleen cells (S) which have formed rosettes with sheep erythrocytes (E), antibodies to sheep erythrocytes (A), and complement (C) from normal (n) and tumour-bearing (t) mice. Complement from Ehrlich ascites is designated by $C_{as}$. The grey area at the top of the figure represents the range of normal values obtained by reacting $S_n$ with $EAC_n$. Abscissa: Time of collection of $S_t$, $C_t$ and $C_{as}$ in days after tumour grafting.

of anti-E antibody was a 2-mercaptoethanol-sensitive rabbit antiserum to boiled E stroma, which contained a high proportion of IgM to IgG antibody (IgM : IgG=64) as previously described (Clerici *et al.*, 1976).

*Test for rosette-forming cells.*—To determine the percentage of rosette-forming cells, 0·15 ml of 1% suspension of the EACn or EACt indicator was mixed with an equal amount of a suspension of normal (Sn) or "cancerous" (St) spleen cells ($5\times10^6$/ml), centrifuged at 200 *g* for 5 min and incubated, without dispersing the pellet, at 37°C for 30 min. The cell pellet was then gently dispersed with a Pasteur pipette and the percentage of rosette determined in a haemocytometer by counting about 1000 cells. A lymphocyte was scored as rosette-forming if 3 or more erythrocytes adhered. Normal (Sn) and cancerous spleen cells isolated at the time of killing (St3–St15) were incubated with EACn and EACt3–EACt15 indicators, or with EACas5–EACas15.

*C3b-inhibitor assay.*—In a total volume of 1 ml, the EACn indicator was incubated with a 10% final concentration of normal or tumour-bearing serum collected 7 days after AT grafting, heated at 56°C for 30 min (complement heat-inactivated=CnH56 or CtH56). After 30 min at 37°C, the cells were washed ×3 in HBSS and resuspended to a concentration of 1% in HBSS. The EACn/CnH56 and EACn/CtH56 indicators were used in the rosette test with Sn and St3–St15 cells, as described above.

### RESULTS

### *Rosette formation with serum complement*

As shown in Fig. 1, the number of rosettes obtained by using the $EAC_{t3}$–$EAC_{t15}$ indicators is inversely proportional to the time elapsed from the i.p. injection of AT cells and, one week after tumour grafting, the number of rosettes is about 20% of that found in normal controls.

There were no significant differences between $S_{t3}$–$S_{t15}$ and $S_n$ cells, since the slopes of the curves of the rosette-forming cells obtained for each killing time by incubating the $EAC_{t3}$–$EAC_{t15}$ indicators with $S_n$ or $S_{t3}$–$S_{t15}$ cells were superimposable. Furthermore, the numbers of rosettes

FIG. 2.—The columns show the number of RFC/$10^6$ $S_n$ or $S_t$ with $EAC_n$ preincubated with 56°C-heated serum from normal ($C_nH56$) or tumour-bearing ($C_tH56$) mice as a source of C3b inactivator. $C_nH56$ and $C_tH56$ are the abbreviated notations for $EAC_n/C_nH56$ and $EAC_n/C_tH56$. Other symbols as in Fig. 1.

formed by $S_n$ or $S_{t3}$–$S_{t15}$ with the $EAC_n$ indicator were the same.

*Rosette formation with ascites complement*

The results obtained using the $EAC_{as5}$–$EAC_{as15}$ indicators (also reported in Fig. 1) show that: (1) $S_n$ cells form fewer rosettes when incubated with $EAC_{as5}$–$EAC_{as15}$ than with $EAC_{t3}$–$EAC_{t15}$ in the early period of tumour growth (3–5–7 days); and (2) the number of $EAC_{as}$ rosettes equals that of $EAC_t$ rosettes 10–15 days after the i.p. injection of AT cells.

*Rosette formation after incubation of $EAC_n$ indicator with C3b-inactivating enzyme*

As shown in Fig. 2, the number of rosettes formed with $EAC_n$ indicator incubated with normal or tumour-bearing heat-inactivated sera as source of C3b-inactivating enzyme, is higher when $S_n$ rather than $S_{t3}$–$S_{t15}$ cells are employed, independently of the use of an $EAC_n$/$C_nH56$ or an $EAC_n/C_tH56$ indicator. In other words, the difference between sera is not statistically significant, while between cells it is ($P<0{\cdot}05$).

DISCUSSION

Normal ($S_n$) and cancerous spleen cells isolated at the time of killing ($S_{t3}$–$S_{t15}$) were incubated with $EAC_n$ and $EAC_{t3}$–$EAC_{t15}$ indicators in order to check at the same time the capacity of $C_t$ to react with the EA complex, and the variations, if any, of the number of cellular receptors for $EAC_t$ and $EAC_n$.

Results, summarized in Fig. 1, show that activity of cancerous serum as source of complement ($C_t$) is inversely proportional to the time from the i.p. injection of Ehrlich ascites carcinoma cells. One week after tumour grafting, the number of rosettes is about 20% of that found in normal controls.

The decrease is not caused by modification of the cellular membrane of the lymphocytes of the AT-bearing mice, since there are no significant differences between $S_{t3}$–$S_{t15}$ and $S_n$ cells. In fact the slopes of the curves of the rosette-forming cells obtained for each killing time by incubating the $EAC_t$ indicators with $S_n$ or $S_{t3}$–$S_{t15}$ cells were superimposable. Furthermore, the numbers of rosettes formed by $S_n$ or $S_{t3}$–$S_{t15}$ with $EAC_n$ indicator were the same, thus showing that the total amount of spleen cell receptors for complement was not modified during tumour growth. The experiments performed by treating the $EAC_n$ indicator with the C3b-inactivating enzyme, which splits C3b into C3d, which remains complex-bound, and 2 minor peptides which are released in the medium, show that: (a) serum from cancerous mice contains a normal concentration of the enzyme; and (b) Blymphocytes from the same animals have a normal amount of C3d receptors. It is known that macrophages have receptors only for C3b, while B lymphocytes possess also those for C3d, which arises through the action of C3b-inactivating enzyme. Our results (Fig. 2) show that the number of $S_n$ rosette-forming cells is higher than that obtained with $S_{t3}$–$S_{t15}$ cells, independently of the use of an $EAC_n/C_nH56$ or an $EAC_n/C_tH56$ indicator. In other words the difference between sera is not statistically significant, while that between cells has $P<0{\cdot}05$. Such a difference may indicate that B lymphocytes of cancer-bearing mice have fewer C3d receptors than those of normal mice. However, it is worth remembering that the rosette assay has been performed with spleen-cell suspension containing both lymphocytes and macrophages. A relative increase of the number of macrophages in the spleen of cancer-bearing mice could explain the decreased number of rosettes formed after incubation of spleen cells with $EAC_n$ complexes pretreated with C3b-inactivating enzyme (CnH56 or CtH56) so that the stable C3d site becomes accessible to cells which carry C3d receptors, like B lymphocytes. Indeed, previous observations by ourselves showed that the number of macrophages contained in the spleen of mice grafted with AT cells is significantly increased above controls (Clerici *et al.*, 1976). In view of these findings, the smaller number of tumoral spleen cells rosetting with EACn/CnH56 (or EACn/CtH56), that is, with EAC3d complexes, can reasonably be correlated with the relative increase in the number of spleen macrophages brought about by the growth of the transplanted tumour. Therefore, it may be deduced that Swiss mice bearing the AT cells lack the complement factors responsible for immunoadherence, while the cellular receptors for such factors are unaffected by tumour growth.

It is difficult to define the mechanism of this complement depletion and to determine whether it may affect the antitumoral reactivity of cancerous mice. If a hypothesis can be formulated, it is possible that complement is consumed through the alternative pathway which is activated during the inflammatory responses accompanying the tumour growth. The alternative pathway represents the ancestral complement pathway, which can be activated as a first line of defence through several different and non-immune mechanisms. This hypothesis is supported by measurement of the complement level in the ascitic fluid ($C_{as}$) performed by using the $EAC_{as5}$–$EAC_{as15}$ indicators in the rosette method. The results (Fig. 1) show that: (1) $S_n$ cells form fewer rosettes when incubated with ascites than with AT-bearing serum in the early period of tumour growth (3–5–7 days); and (2) the number of $EAC_{as}$ rosettes equals that of $EAC_t$ rosettes 10–15 days after the i.p. injection of Ehrlich ascites tumour cells.

It is possible that this behaviour is the result of an excessive complement consumption at the site of tumour implantation during the most active multiplication of malignant cells, and the cancer-bearing mice are unable to synthesize new complement in an amount sufficient to keep its concentration constant in the body fluids, as suggested by other authors (Kassel *et al.*, 1973; Irie, Irie and Morton, 1974; Segerling, Ohanian and Borsos, 1976; Bentley *et al.*, 1976).

### REFERENCES

Baltch, A. L., Osborne, W., Bunn, P. A., Canarile, L. & Hassirdjian, A. (1960) Serum Complement and Bacteriophage Neutralization Titers in Human Infections, Leukemias and Lymphomas. *J. lab. clin. Med.*, **56**, 594.

Bentley, C., Bitter-Suermann, D., Hadding, U. & Brade, V. (1976) *In vitro* Synthesis of Factor B of the Alternative Pathway of Complement Activation by Mouse Peritoneal Macrophage. *Eur. J. Immun.*, **6**, 393.

Bourdin, J. S., Saracino, R. T., Lille, I. & Gabay, P. (1964) Etude des Variations du Taux du Complement au Cours de la Chimiothérapie des Cancers. *Acta Un. int. Cancr.*, **20**, 411.

Clerici, E., Bigi, G., Garotta, G., Porta, C. & Mocarelli, P. (1976) T-cell Precursors in Mice Bearing the Ehrlich Ascites Tumors. *J. natn. Cancer Inst.*, **56**, 513.

Gewurtz, H. & Suyehira, L. A. (1976) Complement. In: *Manual of Clinical Immunology*, Eds. N. R. Rose and H. Friedman. American Society for Microbiology, Washington, p. 36.

Hartveit, F. (1964) The Complement Content of the Serum of Normal as Opposed to Tumour Bearing Mice. *Br. J. Cancer*, **18**, 714.

IRIE, K., IRIE, R. F. & MORTON, D. L. (1974) Evidence for *In vivo* Reaction of Antibody and Complement to Surface Antigens of Human Cancer Cells. *Science, N.Y.*, **186**, 454.

KASSEL, R. L., OLD, L. J., CARSWELL, E. A., FIORE, N. C. & HARDY, W. D., JR (1973) Serum Mediated Leukaemia Cell Destruction in AKR Mice. Role of Complement in the Phenomenon. *J. exp. Med.*, **138**, 925.

LICHTENFELD, J. L., WIERNIK, P. H., MARDINEY, M. R., JR & ZARCO, R. M. (1976) Abnormalities of Complement and its Components in Patients with Acute Leukemia, Hodgkin's Disease and Sarcoma. *Cancer Res.*, **36**, 3678.

MASON, D. W. (1976) The Requirement for C3 Receptors on the Precursors of 19S and 7S Antibody Forming Cells. *J. exp. Med.*, **143**, 1111.

SEGERLING, M., OHANIAN, S. H. & BORSOS, T. (1976) Persistence of Immunoglobulin and Complement Components C4 and C3 Bound to Guinea Pig Tumor Cells. *J. natn. Cancer Inst.*, **57**, 145.

SILVEIRA, N. P., MENDES, N. F. & TOLNAI, M. E. (1972) Tissue Localization of Two Populations of Human Lymphocytes Distinguished by Membrane Receptors. *J. Immun.*, **108**, 1456.

SOUTHAM, C. M. & SIEGEL, A. H. (1966) Serum Levels of Second Component of Complement in Cancer Patients. *J. Immun.*, **97**, 331.

TING, C. C. & HERBERMAN, R. B. (1976) Humoral Host Defence Mechanisms against Tumors. *Int. Rev. exp. Pathol.*, **15**, 93.

VERHAEGEN, H., DECOCK, W., DECREE, J. & VERGRUGGEN, F. (1976) Increase of Serum Complement Levels in Cancer Patients with Progressing Tumors. *Cancer, N.Y.*, **38**, 1608.

YOSHIKAWA, S., YAMADA, K. & YOSHIDA, T. O. (1969) Serum Complement Level in Patients with Leukemia. *Int. J. Cancer*, **4**, 845.

ZARCO, R. M., FLORES, E. & RODRIGUEZ, F. (1964) Serum Complement Levels in Human Cancer. *J. Philipp. med. Ass.*, **40**, 839.